# Aspects of Gene Therapy Products Using Current Genome-Editing Technology in Japan

**DOI:** 10.1089/hum.2020.156

**Published:** 2020-10-16

**Authors:** Teruhide Yamaguchi, Eriko Uchida, Takashi Okada, Keiya Ozawa, Masafumi Onodera, Akihiro Kume, Takashi Shimada, Satoru Takahashi, Kenzaburo Tani, Yasutomo Nasu, Tomoji Mashimo, Hiroyuki Mizuguchi, Kohnosuke Mitani, Kazushige Maki

**Affiliations:** ^1^ Kanazawa Institute of Technology, Ishikawa, Japan; ^2^ Nihon Pharmaceutical University;; ^3^ National Institute of Health Sciences;; ^4^ The University of Tokyo;; ^5^ Jichi Medical University;; ^6^ National Center for Child Health and Development;; ^7^ Nippon Medical School;; ^8^ University of Tsukuba;; ^9^ Okayama University Graduate School of Medicine, Dentistry and Pharmaceutical Sciences; ^10^ Osaka University;; ^11^ Saitama Medical University;; ^12^ Pharmaceuticals and Medical Devices Agency.

**Keywords:** gene therapy, genome editing, off-target effect, on-target mutagenesis, safety, double-strand break

## Abstract

The development of genome-editing technology could lead to breakthrough gene therapy. Genome editing has made it possible to easily knock out or modify a target gene, while current gene therapy using a virus vector or plasmid hampering modification with respect to gene replacement therapies. Clinical development using these genome-editing tools is progressing rapidly. However, it is also becoming clear that there is a possibility of unintended gene sequence modification or deletion, or the insertion of undesired genes, or the selection of cells with abnormalities in the cancer suppressor gene *p53*; these unwanted actions are not possible with current gene therapy. The Science Board of the Pharmaceuticals and Medical Devices Agency of Japan has compiled a report on the expected aspects of such genome-editing technology and the risks associated with it. This article summarizes the history of that discussion and compares the key concepts with information provided by other regulatory authorities.

## Introduction

In the past decade, advances in genome-editing technologies^1,2^ have provided the possibility of a dramatic improvement in gene therapy not only *in vitro* but also *in vivo*. Genome editing is a technology that can bring about either the knockout (KO) of a target gene or the homologous recombination of genes of interest (homology-directed repair [HDR]) through double-strand breaking (DSB) of a target DNA sequence. The targeting ability of genome-editing enzymes is dependent on the recognition by specific protein to be able to bind target DNA sequence (using a zinc-finger nuclease [ZFN],^3^ or a transcription activator-like effector nuclease [TALEN]^1^ or by binding single-guide RNAs [sgRNAs], which form clustered regularly interspaced short palindromic repeats [CRISPR]/CRISPR-associated proteins [Cas] protein complexes^4^).

These genome-editing nucleases are able to cause a DSB in the target DNA sequence, and the targeted gene knockout could then be induced by a frame shift-induced mutation in generating DSBs during the repair process in a cell.^3^ Genome-editing tools may also be utilized to induce homologous recombination with donor DNA fragments, resulting in modification of the hereditary disease gene with normal gene. Therefore, to effectively apply genome editing, it is critical to have a design that will introduce the DSB into the appropriate locus in the target gene sequence. Current gene therapy products using lentiviral or retroviral vectors that deliver a specific gene to introduce patient cells can integrate the gene of interest into patient cell chromosomes, but cannot yet target the gene into any specific chromosomal locus. Other viral vectors such as adenovirus or adeno-associated virus (AAV) have no genome integration machinery but may be integrated into nonspecific sites, although with extremely low efficiency. Thus, the targeting ability of genome-editing technologies provides new potentials for elimination-(knockout) or insertion of specific genes or the repair of damaged genes and the promise of “correcting” genetic disease or as novel treatments for infectious diseases.

Although no genome-editing gene therapy product has yet been approved worldwide, many clinical study protocols using genome-editing tools have been approved and are ongoing. The advantageous aspects of genome editing are utilized to exploit either knockout technology for a target gene by nonhomologous end joining or the modification of hereditarily abnormal genes with normal ones by HDR. In contrast, genome-editing technologies have been known to cause DSB on nontarget genes (off-target effect), or to yield insertions and deletions (indels), or templated repair from a separate donor DNA molecule (on-target mutation). To avoid off-target effects, genome-editing technologies that do not cause DSB, such as base editing and death editing, have been explored. New analytical methods and new technologies continue to be developed to combat the various undesirable effects of genome editing.

The Science Board of the Japan Pharmaceuticals and Medical Devices Agency has established an Expert Committee for Genome Editing to discuss the quality and safety issues of these technologies. In early 2020, the committee published its white paper on the evaluation of genome editing to educate both industry and regulatory scientists. In this article, we discuss the key points of the white paper and compare them with information provided by other regulatory agencies as follows:

1. All information with respect to classifications; CLASSIFICATION OF GENOME-EDITING PRODUCTS FOR GENE THERAPY & ISSUES IN SPECIFIC GENOME EDITING COMPARED WITH THOSE OF CURRENT GENE THERAPY PRODUCTS
2. All quality aspects with respect to product development; Cautions regarding genome-editing tools and gene-modified cells
3. All kinetics and safety aspects with respect to product development; Classification by purpose of genome editing & SAFETY EVALUATION
4. All clinical aspects with respect to product development; IMPORTANT ISSUES IN CLINICAL TRIALS (INCLUDING LONG-TERM FOLLOW-UP)

## Classification of Genome-Editing Products for Gene Therapy

The white paper first classifies genome-editing products according to the editing techniques and technologies (tools) that introduce them into a cell or a specific tissue in the body, and then discusses the purposes of genome editing to clarify the characteristics of each technique/technology. Genome-editing products are categorized into three types depending on the method used to express or introduce genome-editing enzymes into cells, as shown in Table 1.

**Table 1.** Definition of genome-editing products

• *In vivo* genome-editing products (gene therapy products that use at least one genome-editing technology and that are administered directly in the body)
➢ Gene therapy vector products for genome editing (gene therapy products consisting of a viral or plasmid vector that expresses desired proteins, that is, nucleases used for genome editing) and gRNAs for genome editing
➢ mRNA products for genome editing (mRNA that expresses desired proteins for genome editing)
➢ Protein products for genome editing (desired proteins or protein/gRNA complexes used for genome editing)
• *Ex vivo* genome-editing products (human cell-based products genetically modified by a genome-editing tool)

gRNA, guide RNA; mRNA, messenger RNA.

Early clinical studies made use of adenovirus,^5^ adeno-associated viral vectors,^6^ or plasmids^7^ to express genome-editing enzymes, and mRNAs^8^ coding ZFNs or TALENs were also used. The direct introduction of genome-editing enzymes to modify a target gene have also been explored in both *ex vivo* and *in vivo* treatments.

## Issues in Specific Genome Editing Compared with those of Current Gene Therapy Products

### Risk from undesired genome modification and cancer risk

Genome-editing technologies provide novel tools to replace, eliminate, or modify target genes in a DNA sequence-specific manner. These technologies are expected to provide novel and valuable products that will make it possible to insert, delete, or replace desired DNA in human genomes using engineered site-specific nucleases. However, genome editing has the inherent risk of unintended editing of genes that have similar DNA sequences; this is known as the off-target effect. In addition to the risk of unintended gene modification, the long-term effects of off-target genome editing remain unknown. Of particular concern is that off-target effects may result in tumorigenicity (cancer). Off-target effects may directly activate oncogenes or inactivate tumor-suppressor genes. Importantly genetic modifications by genome editing have the potential to cause permanent alterations in genomes.

It has been also reported that genome-editing techniques that induce DSBs may induce genome instability, which is associated with chromosomal breaks. With some technical limitations, undetectable large-scale defects and the insertion of DNA sequences into DSB sites in chromosomal sequences that cannot be detected by current analytical technologies have also been reported.^9^ These undesired modifications of chromosomes may be caused not only in on-target sites but also in off-target sites. The potential risks that stem from these chromosomal aberrations due to undesired modification of chromosomes, including tumorigenicity, should be assessed preclinically.

### Risk of unintended gene modification in germline cells

*In vivo* genome editing where a genome-editing gene therapy product is administered directly to the patient may unintentionally result in genome editing of unintended cells or the modification of off-target genes. The fact that it is difficult to identify and eliminate these unintended alterations of cells and genes when they occur is a key safety concerning *in vivo* genome editing.

Of particular concern is the fact that *in vivo* genome editing for pediatric patients and patients of reproductive age may affect germline cells. Possible genetic effects in subsequent generations should be fully elucidated.

To avoid the risk of chromosomal mutations attributable to genomic cleavage, new technologies that allow genetic engineering without genomic cleavage have recently been developed. For the *in vivo* application of these new types of genome editing, the effects on the next generation should be evaluated through nonclinical and clinical studies.

## Classification of Genome-Editing Technologies and Challenges Related to their Characteristics

Many genome-editing methods and related technologies have been and continue to be developed, and suitable technologies from the genome-editing toolbox can now be selected. Gene therapies to specifically modify or knock out a target gene were much desired in the early phases of the development of gene therapy. Until genome-editing technologies were developed, it was very difficult to make such modifications. Let us now consider the characteristics of genome-editing technologies.

### Classification by genome-editing tool and points to consider

#### ZFN and TALEN

ZFNs are artificial nucleases engineered by fusing a domain that contains three to six zinc-finger protein motifs that recognize three specific base pairs and bind a targeted DNA sequence to a DNA-cleavage enzyme *FokI* nuclease (*Fok*I).^3^ Since designing this DNA-binding protein requires highly sophisticated technologies, ZFNs have not been utilized as a genome-editing tool by many researchers. In contrast, TALENs were developed to simplify the complicated ZFN design process.^1^ In TALENs, each TAL module consists of 34 amino acids of TAL, a plant-derived transcription factor, and each module recognizes one nucleotide. By binding four different types of TAL module, each of which recognizes nucleotide base A, G, C, or T, targeted DNA sequences can be recognized so that specific DNA sequences can be cut by *Fok1* nuclease, which is fused to TAL modules. Typically, the TALs are designed to recognize 15–20 nucleotides by binding 15–20 TAL modules.

Since the *Fok*I domains of ZFNs and TALENs cut only a single chain of DNA double strands, two artificial enzymes that recognize DNA sequences upstream and downstream of the target cleavage site are required to make a DSB on a target sequence. A recognition sequence of 18–40 bases is required to form one DSB, which is twice the length of the sequence recognized by one artificial enzyme. This makes the target DNA recognition specificity of both ZFNs and TALENs high. TALENs are thought to be less likely to cause off-target effects than CRISPR/CRISPR-associated protein 9 nuclease (Cas9).^1,2^

#### CRISPR/Cas

A novel genome-editing tool based on bacterial Cas9 from *Streptococcus pyogenes* has been reported.^10^ Unlike ZFNs and TALENs, a sgRNA complementary to the target DNA sequence is responsible for the recognition of specific DNA sequences in CRISPR/Cas. The sgRNA, therefore, could easily be designed to have a guide sequence that complementarily binds to 20 target nucleotides of the target DNA sequence and a proto-spacer adjacent motif adjacent to the target sequence.

The sgRNA and Cas9, an enzyme that cleaves the double strands of DNA, together form a complex to cleave a gene having the sequence recognized by the sgRNA. It has been noted that the sgRNA binds to the target DNA sequence even in the presence of up to five base pair mismatches (base pairs other than A:T and G:C). The risk of this CRISPR/Cas system inducing off-target effects, such as undesired insertions and deletions of DNA in off-target sequences, will thus be quite high.^6^ Although many reports on off-target effects caused by CRISPR/Cas have been published,^11–13^ there is as yet no standard method. It is, however, difficult to characterize or detect the exact off-target effects that occur rarely, particularly those seen in only a small number of cells.

To mitigate the off-target effects of CRISPR/Cas, the effects of the length of the guide RNA or the second structure of the target DNA sequence have been investigated. However, not enough resolution for this has yet been established. Therefore, given the results of the off-target analysis described as follows, it is essential to design an sgRNA based on the latest knowledge and evaluate the frequency of off-target effects.

#### Genome editing without genomic breaking

To avoid undesirable effects due to the genome instability resulting from a DSB, genome editing without genomic breaking (*e.g.*, single-base editing with deaminase or dead Cas) and other editing technologies have been developed.^14,15^ Although there are many new genome-editing technologies that do not cause DSB, they may potentially modify the genome sequence at sites other than the target loci. For each newly developed genome-editing technology, mechanisms to reduce off-target effects should be justified together with the evaluation methodology.

### Cautions regarding genome-editing tools and gene-modified cells

As described earlier, there are several methods for introducing genome-editing tools into the target cells/tissues both *in vivo* and *ex vivo*. Based on the characteristics of different transfection methods, it is important to select the most suitable method to achieve the desired clinical effect.

#### Viral vectors and plasmid vectors

According to the information on the National Institutes of Health (NIH) ClinicalTrial website, many clinical trials of viral vectors, including adenovirus, AAV and others, have been conducted to introduce ZFN or CRISPR/Cas into a cell for genome-editing gene therapy.^6,16^ Quality control measures for current gene therapy products can also be applied to gene therapy using viral or plasmid vectors^17^ coding a genome-editing enzyme gene. For the past three decades, ∼4,000 clinical protocols for gene therapy have been conducted or are ongoing worldwide (NIH ClinicalTrials.gov). There has been sufficient clinical experience of gene therapy using viral or plasmid vectors for several gene therapy products using AAV, lentivirus, or retrovirus vectors to have been approved for market authorization, and many guidelines for quality, safety, and efficacy have been published. The quality control and characterization for the manufactured vectors, and the establishment and characterization of cell bank systems, should be evaluated in the same manner as current gene therapy products.

In many cases, viral promoters are utilized to achieve efficient expression of the target protein. However, the insertion of viral promoter sequences adjacent to a cancer-related gene could possibly cause tumorigenicity.^12^ In some cases, genome editing also makes use of viral promoters to express ZFNs, TALENs, or Cas9/sgRNA. To the best of our knowledge, oncogenesis by promoter insertion has not yet been reported, although a transfected plasmid DNA has been integrated into the DSB site. Genome-editing gene therapy products using a viral or plasmid vector require nonclinical safety evaluation similar to those used to evaluate conventional gene therapy products. The cells or tissue tropisms should also be assessed together with their biodistribution.

When a viral vector is used to introduce a genome-editing tool into a cell, the extent and frequency of the gene modification of the target cell and off-target cells should be analyzed from the viewpoints of infectivity and cell tropism. It should also be taken into account that the persistent expression of a genome-editing enzyme can increase the possibility of off-target effects, especially since viral vector-mediated genome editing can cause the long-term persistent expression of the genome-editing enzyme. The persistent expression of genome-editing enzymes should be evaluated from the viewpoint of safety.

#### mRNA

To express genome-editing proteins such as Cas, TALEN, and ZFN, intracellular transfection of the mRNA that codes for these proteins has been utilized.^16,18,19^ In accordance with the Act on Securing Quality, Efficacy and Safety of Products Including Pharmaceuticals and Medical Devices (PMD Act), mRNA products are defined as “Gene expression products for treatment” as one kind of gene therapy product. Although mRNA-based products have also been developed in fields other than genome editing, the guidelines for the Quality and Safety Assurance of Gene Therapy Products do not cover the quality or safety of mRNA. Currently, however, no mRNA product has yet been approved for marketing in Japan or overseas. The manufacturing method and quality control of mRNA-based products remain to be clarified in the future. In particular, gene editing using mRNA manufactured by chemical modification such as methylated Cap, which does not occur naturally, to ensure the intracellular stability of mRNA also requires safety evaluation of the applied chemical modification. Sponsors are encouraged to engage in full consultation with the relevant regulatory authorities about the quality control and safety evaluation of mRNA. The same product management methods that are applied to nucleic acid products can also be applied to chemically synthesized mRNA products. mRNA synthesized by *in vitro* transcription using a plasmid or PCR product as a template should be required to undergo additional safety evaluation for process-derived impurities such as RNA polymerase or template DNA.

#### Proteins and guide RNA

Both ZFN and TALEN proteins consist of two components: a DNA binding domain and a DNA cleavage domain (*Fox1* nuclease).^20,21^ The design of the DNA binding domain is the key element of genome-editing enzymes; for example, either the specificity of the target DNA or the efficiency of these artificial nucleases. A CRISPR/Cas technique can be conducted to introduce a complex (ribonucleoprotein) of sgRNA complementary to the target DNA sequence and Cas9 into a cell.^22,23^ The specificity of CRISPR/Cas depends primarily on the design of the sgRNA sequence. As a genome-editing technology, the direct transfection of these genome-editing enzymes could cause DSB on the target sequence in the cells, resulting in KO or HDR in the target gene. Therefore, protein-based genome editing also carries a risk of either off-target effects or adverse events and there may also be potential on-target mutations in the target sequences accompanied by DSB (undesired large deletions or insertions of DNA).^24^ In this sense, genome-editing products need to be evaluated as gene therapy products as with the current products used for gene transfer technologies.

In contrast, the quality of artificial nuclease protein products such as ZFN, TALEN, and CRIPR/Cas should be evaluated according to the International Conference on Harmonization (ICH) biotechnology guidelines for evaluation and quality control of cell banks for biotechnological products (ICH Q6B).

#### Human cell-based products modified by genome-editing technology

The quality control strategy for human cell-based products manufactured from current gene-transfected cells can be applied to human cell-based products manufactured from *ex vivo* genome-edited cells. The quality control and characterization for the manufacture of vectors, and the establishment and characterization of cell bank systems should be evaluated in the same manner, that is, as per current ICH guidelines (ICH Q5A, Q5B, and Q5D). To assure the safety of administering genome-edited cell-based products, nonclinical safety assessment similar to that for human cell-based products made from conventional gene-transfected cells is essential.

### Classification by purpose of genome editing

#### Gene knockout^25–30^ and homologous recombination^11,31,32^

When gene editing is intended to knock out genes, the frequency of gene knockouts in the target cells and the heterogeneity of targeted gene modifications should be analyzed. For example, in CRISPR/Cas-based gene editing, a justification of the sgRNA design should include conclusions on efficiency and heterogeneity in target cells. Although gene editing by HDR is based on the DSB repair mechanism of cells, it has been reported that the activity of homologous recombination was found to be high in embryonic stem (ES) cells, which have a high ability to repair DSB.^33^ It should be noted that the DSB repair efficiency can be very low in some types of cells. Therefore, homologous recombination for the development of gene-editing products intended to cause HDR on target genes should also be clarified and justified for clinical efficiency. It might be supposed that the efficiency of HDR in target cells will be very low.^31,34^ In this case, it might be necessary to select and purify gene-modified cells that have undergone homologous recombination for treatment, and the methods for cell selection/purification should then be justified.

Homologous recombination is required to introduce the donor DNA for the modification of the target gene. To modify short DNA sequences such as single-nucleotide polymorphisms,^33^ single-strand DNA that has a homologous sequence both upstream and downstream of the DSB site is used to induce homologous recombination. In HDR to replace the entire gene coding for a protein, a plasmid is typically used as a donor DNA template. In this case, DNA with a homologous sequence of several hundred DNA sequences from upstream to downstream of the DSB should be introduced to the target site. Therefore, it is crucial to evaluate the design of the donor DNA and the efficiency of homologous recombination. Even though some reports suggest that there is no correlation between a DNA length that can undergo homologous recombination and recombination efficiency, homologous recombination efficiency should be evaluated together with the effects of the genome length.^35,36^

For either simultaneous knockouts of more than one gene or more efficient homologous recombination, gene editing involving two DSBs has been explored. The introduction of two or more DSBs is likely to be associated with significant chromosomal aberrations such as chromosomal translocations and/or deletions. In these cases, chromosomal aberrations should be fully evaluated.^37^

#### A new technology such as gene modification without genomic cleavage (DNA modification without DSB such as Dead Cas9, deaminase, or DNA methylation/demethylation)

**Figure 1.**
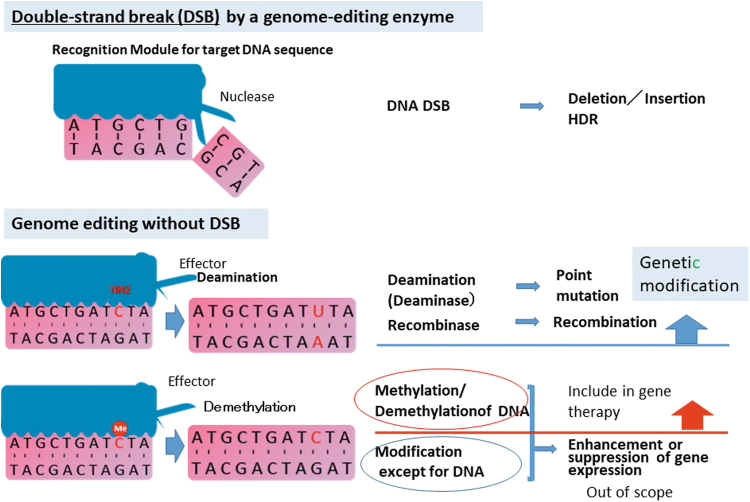
Definition and categories of genome editing.

To prevent chromosomal breaks, translocations, and large deletions associated with genome editing, genome-editing technologies without DSB have been developed^38–40^ as a new approach to genome editing that enables the direct irreversible conversion of one target DNA base into another in a programmable manner, such as the conversion of C to T or A to G with a deaminase.^41,42^ In addition, genome editing to introduce epigenetic mutations in target sequence, such as DNA methylation, is actively being developed. However, these genome-editing technologies without DSB may also cause adverse events attributable to off-target effects on untargeted genes. Genome-editing products for human diseases should be regulated as gene therapy products, and many types of products have been developing, for example, products that cause KO or HDR of target genes that cause diseases, deadCas, and baseEditing. Recently, genome editing has been developed that does not alter DNA, but enhances the target gene through the modification of histone or merely binding to the target gene (Fig. 1). Since the efficacy or specificity of based gene engineering without the induction of DSB may be different in each cell, selection and/or purification of gene-modified cells may be necessary. Therefore, quality assessment of techniques without DSB should be conducted based on these assumptions. In addition, the adequacy of each genome-editing technology must be determined using the optimal analysis technique according to the nature of the technology.

## Safety Evaluation

### Issues in the application of gene therapy products using genome-editing techniques

For the safety of genome-editing technologies, many issues associated with genome-editing nucleases should be considered, such as off-target mutations and unwanted chromosomal translocations associated with off-target and on-target DNA cleavages. Regarding the application of genome editing to induce HDR, the mutation of p53 protein should be analyzed. Furthermore, since genome-editing enzymes are derived from nonhuman proteins, the risk of an immune reaction to the genome-editing enzyme must also be assessed (Table 2).

**Table 2.** Safety issues in gene therapy products using genome-editing techniques

• Off-target effects
• On-target mutagenesis
• Chromosomal changes (translocation/inversion/deletion)
• *P53* mutation
• Immunogenicity of nucleases
• Germline modification

#### Off-target effects

**Figure 2.**
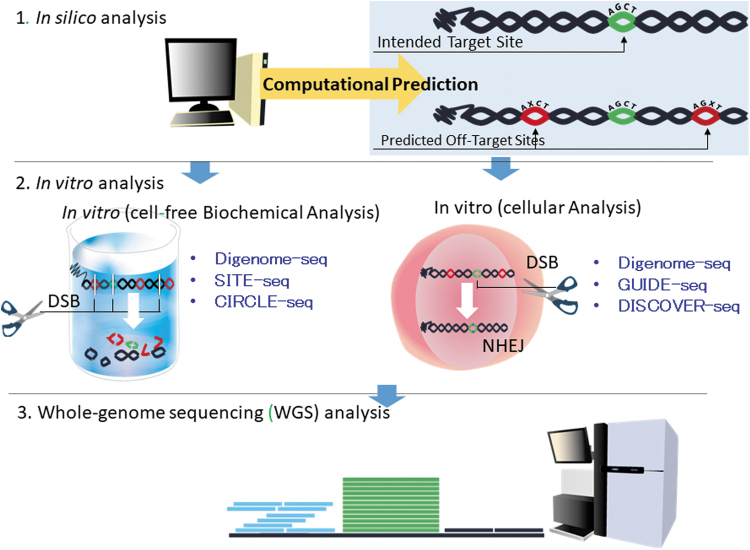
Analysis of off-target effects.

To characterize the off-target effects of genome-editing gene therapy products, it is necessary not only to predict the existence of sequences similar to the target gene sequence by *in silico* analysis, but also to experimentally explore candidate off-target sites throughout the entire human genome (Fig. 2).^43–46^ Such off-target profiling methods include GUIDE-seq,^47^ which involves the introduction of a tag of synthetic DNA in the cleavage site for genome-wide sequencing of the tag, and DIGENOME-seq,^48,49^ CIRCLE-seq,^50^ and SITE-seq,^51^ which explore potential off-target cleavage sites of genome-editing enzymes using genome DNA extracted from cells. These analyses will provide information focused on mutations such as SNV/Indel and the copy number variation of cancer-related genes.^52^ It should be determined whether breaks or deletions have actually occurred at the off-target sites predicted by *in silico* analysis, and experimental methods should be evaluated based on the whole genome sequence^44,46^ of the genome-edited cell and amplicon sequence, which involves PCR amplification of candidate off-target sites followed by deep sequencing.^53^ It should be noted that although the detection sensitivity of these analyses depends on the read depth of DNA sequencing, it is very difficult to detect off-target effects that occur with a frequency of 0.1% or less. If the results of characterization of an *ex vivo* genome-editing-based product show off-target effects of the gene editing, the risk of those off-target effects causing cancer or other adverse events should be evaluated. Clonality analysis such as linear-amplification-mediated PCR of gene-modified cells may be required.

To ensure the safety of genome-editing technology, it is necessary to mitigate off-target effects. The design of sgRNA may be the most critical factor in mitigating the off-target effects of CRISPR/Cas. It is also important to use *in silico* analysis to select DNA sequences with few homologous sequences in other genomic regions; however, it must be noted that *in silico* analysis may not be able to predict all candidate off-target sites. A combination of *in silico* and *in vitro* analyses is useful in identifying candidate off-target sites, and it is very important to understand the frequency of off-target effects and their influence. Since in *in vitro* analysis, natural gene mutations may occur in cells during culture, such background mutations should be excluded in assessing gene mutations associated with genome-editing procedures.

In the case of *in vivo* genome editing, an analysis of the off-target effects of genome editing in animals as a nonclinical study is not appropriate because of species differences in the genome sequence between humans and animals. As part of the characterization studies of *in vivo* genome editing, therefore, the frequency of off-target events and affected DNA sequences should be analyzed in detail in *in vitro* assays using human cells. To evaluate off-target effects on *in vivo* genome-editing products, *in vitro* analysis using a continuous cell line harboring many DNA mutations may not provide useful data. To assess the off-target effects of *in vivo* genome editing, analysis using primary cells is recommended, and induced pluripotent stem (iPS) cells or ES cell-derived functional cells may also be useful. iPS and ES cell-derived cells are very promising tools for the assessment of off-target effects on human primary cultured cells that are not easily available.

#### Genome deletions/insertions of unintended DNA sequences and chromosomal translocations/inversions

It has been reported that large deletions (of several kb), and insertions and inversions of gene fragments during the DSB repair process may occur during genome editing (on-target mutagenesis).^54^ The insertion in the target site of the genome DNA of a viral vector used for genome editing has also been reported.^55,56^ This may occur because gene modifications by genome editing depend on the DSB-induced genome repair mechanism of cells in which the genome-repairing mechanism could not be controlled by genome-editing technology. The directionality of the genome editing how to modify or repair the target genome is not involved in the genome-editing tool.^54^ Therefore, the target gene and/or its flanking genome sequence in cells/tissues as similar as possible to the actual target cells should be analyzed in detail. As mentioned earlier, the risk of chromosomal translocations and deletions after a DSB has been reported.^57–59^ Such chromosomal aberrations should be analyzed using G-band analysis, Q-band analysis, multicolor fluorescent *in situ* hybridization (mFISH) using false colors, comparative genomic hybridization (CGH), and so on. However, it should be understood that these analyses also have certain limitations. For example, G-band analysis and mFISH can be applied to cells only in the metaphase stage. In G-band analysis, it is difficult to deal with many cells at once and detect a very small group of cells having chromosomal aberrations. mFISH is suitable for detecting translocations between different chromosomes and large chromosomal deletions; however, this analysis does not detect inversions within the same chromosome. CGH can detect abnormal gene amplifications and deletions when they occur in many cells, but CGH sensitivity is too low to detect DNA aberrations if they are not uniform across cells or occur only in some cells. In the risk assessment of genome-editing-associated chromosomal aberrations, the characteristics of these analysis methods should be fully taken into consideration.

#### Risk of DNA-repair gene mutations such as *p53* in genome-edited cells

Mutations of the *p53* tumor-suppressor gene in cells in which a gene modification was caused by HDR-based genome editing and an increase in HDR efficiency in cells knocked out for the *p53* gene have been reported.^60,61^ These phenomena can be explained primarily by the fact that *p53* mutations increase resistance to cell death due to genome mutations. The occurrence of gene mutations related to genome repair, such as *p53* mutations, should be investigated in the introduction of HDR-derived genes.

#### Differences in the risk of cancer among target cells

From the point of view of intending to modify genomes, there are common concerns between genome-editing technologies and current gene therapies using chromosomally integrated vectors such as retroviral and lentiviral vectors. From this perspective, the risk of their off-target effects in both products will be similar. From the early phase of the development of gene therapy, the most concerning risk associated with chromosomally integrated vectors was the induction of tumorigenicity in cells due to insertional mutagenesis. In fact, gene therapy using hematopoietic stem cells for the treatment of X-linked severe combined immunodeficiency (X-SCID) or Wiskott–Aldrich syndrome has been reported to cause leukemia.^12^ Currently, therefore, these gene therapies require long-term follow-up to monitor tumorigenesis. Nevertheless, to the best of our knowledge, there have been no reports of cancer caused by gene therapies where a chromosomally integrated vector is introduced into cells other than hematopoietic cells.

Hematopoietic cell-based gene therapies involve the insertion of a vector with a viral promotor/enhancer adjacent to a cancer-related chromosomal gene, which is thought to be the mechanism causing cancer associated with hematopoietic cell-based gene therapies.^62^ Genome editing using neither a promoter nor an enhancer is unlikely to cause inserted mutations to promote cell proliferation. In particular, the direct transfection of a genome-editing tool in the form of a nuclease protein or its mRNA into cells is very unlikely to cause cancer induced by insertional mutagenesis. However, there are other concerns regarding such genome-editing tools, because genome editing may cause chromosomal translocations, deletions, and other aberrations accompanied by DSB. Chromosomal translocations may cause cancer chimeric proteins such as *Bcr-abl* or destroy tumor-suppressor genes.^63^ Genome editing with homologous recombination may cause an increase in the number of cells harboring mutations in tumor-suppressor genes such as p53, as described earlier. The risk of genome editing causing cancer due to chromosomal aberrations or the destruction of tumor-suppressor genes has not yet been fully investigated. Based on reported experience with current gene therapies in which genes are integrated into cells, the risk of carcinogenesis appears to depend on the type of cell. Differentiated cells are likely to be more robust against the risk of carcinogenesis than undifferentiated or stem cells. In contrast, iPS/ES cells and hematopoietic cells have a higher risk of carcinogenesis than other somatic cells.

#### Immunogenicity of genome-editing enzymes

DNA-breaking enzymes used for genome editing, such as Cas protein, are derived from bacteria. Even in *ex vivo* gene therapy, therefore, when genome-edited cells are administered *in vivo* and express these enzymes, the enzymes are recognized as heterologous antigens. The prevalence of pre-existing antibodies to Cas9 has been evaluated, and the prevalence of anti-SaCas9 and anti-SpCas9 antibodies has been reported to be 10% and 2.5%, respectively, in one study using 200 human serum samples^64^; similar results were also obtained in a study by Carsten *et al*.^65^ Immunogenicity in humans may not be predicted from animal studies. Clinical trials should be designed taking into consideration the potential immune toxicity of genome-editing enzymes, that is, the immune response to these enzymes, including the attenuation of clinical effects and anaphylaxis.^66^

#### *In vivo* genome editing

##### Safety evaluation of modified target genes

Where there are safety concerns about the expression of modified target genes, a proof-of-concept study in model animals with the modified homologous gene may provide information about kinetics and safety related to the modification of the target gene. The results of this study should be taken into consideration together with data supporting the efficacy or performance of the gene therapy in question. Careful interpretation of the results obtained in this study due to differences in species.

##### Targeting and modification efficiency of genome-editing enzymes

In *in vivo* genome editing, targeting in the tissues/cells to be modified is important.^67^ It is necessary to characterize the biodistribution of a genome-editing tool or enzyme expressed by the tool *in vivo* to understand its distribution not only to targeted cells/tissues but also to nontargeted cells/tissues. The persistence of the genome-editing enzyme in both desired and undesired tissues/cells should also be elucidated. In particular, when a biodistribution study shows the distribution signal of a genome-editing enzyme in germline cells, the risk of germline gene modification should be clarified in nonclinical studies with reference to the ICH considerations “General Principles to Address the Risk of Inadvertent Germline Integration of Gene Therapy Vectors.”

*In vivo* genome editing may not provide sufficient effects because of its low genome-editing efficiency in target cells/tissues. A variety of technologies to improve efficiency have been actively explored. For example, homology-independent targeted integration is a method in which the same sequence as that at the target DBS site is inversely inserted into the donor vector to cut the genome and the donor vector simultaneously. This technique allows efficient *in vivo* genome editing.^68^ In addition, the introduction of CRISPR/Cas using AAV to express CRISPR/Cas for a prolonged period of time has been reported to enable efficient genome editing of nondividing cells.^69^ However, the long-term expression of genome-editing enzymes such as CRISPR/Cas increases the risk of either off-target effects or undesirable gene modifications at the target sequence. It should be noted that off-target effects and on-target mutagenesis of *in vivo* genome editing, if they occur, are difficult to remove, unlike *ex vivo* genome editing.

##### Other factors

Nonclinical studies using model animals are unlikely to provide useful information about the off-target effects of *in vivo* genome-editing-based products. Limited but somewhat meaningful information about off-target effects may be obtained from *in silico* analysis and *in vitro* analysis using human cells. Clinical development studies for *in vivo* genome-editing technologies should be designed on the basis of the assessment of their potential risks using these analyses. Clinical design should take into account both the identified potential risks and the potential usefulness of each technology or product under consideration.

## Important Issues in Clinical Trials (Including Long-Term Follow-UP)

Since genome-editing technologies are intended to modify target genes permanently, genome editing requires long-term follow-up of patients because these technologies have risks similar to those involved in current gene therapy products using a chromosomally integrated vector. Genome editing, which is used to delete or insert genes at specific sites, could be safer than current gene therapies involving random gene insertions. However, undesired genome modifications accompanied by off-target effects or on-target mutagenesis cannot be excluded. Furthermore, genome editing using homologous recombination may increase the mutation risk of DNA-repair genes such as *p53* and is associated with the risk of chromosomal translocation. To identify adverse events related to these risks, an appropriate follow-up period should be established according to each risk.^70^

The length of the follow-up period should be established based on the specific genome-editing technology used in each case (*e.g.*, gene modification through the direct introduction of a protein or introduction/modification using a viral vector), including the type of target cells, and the targeted gene. Among experiences with current gene-therapy products, the application of genome editing to hematopoietic stem cells in particular has been associated with a high risk of adverse events.^37^ Long-term follow-up plans are strongly recommended, including periodic examinations.

*In vivo* genome editing should be considered to cause gene modification in off-target tissues/cells, especially germline cells. When there is a risk of gene modification in germline cells, measures to prevent that modification from affecting subsequent generations, such as setting an adequate contraception period, should be taken. The risk control measures for genotoxic antineoplastic drugs can help to establish such measures.^71^ Since it is difficult to identify gene mutations in germ cells and fertilized eggs, careful long-term follow-up is required to investigate the off-target effects of *in vivo* genome editing.

## Comparison of the PMDA White Paper with Data from Other Regulatory Agencies

Genome-editing technologies have been progressing rapidly, and national regulatory agencies (NRAs) have published several viewpoints about genome-editing products instead of formal guidelines. Table 3 provides a comparison of the European Medicines Agency (EMA) and US Food and Drug Administration (FDA) viewpoints with those of the Japanese PMDA white paper. The FDA does not define genome-editing products, but the definitions given by the EMA and PMDA are similar. All NRAs assume that genome-editing products include not only viral vectors and plasmids but also mRNA and protein-based products.

**Table 3.** Comparison of regulatory stance for genome-editing products for gene therapy

	US FDA	*EU EMA*	*Japan MHLW/PMDA*
Relevant guidelines, etc.	Chemistry, Manufacturing, and Control (CMC) Information for Human Gene Therapy Investigational New Drug Applications (INDs) (2020.1).	Guideline on the quality, nonclinical and clinical aspects of gene therapy medicinal products (2018.3)	Guidelines for Gene Therapy Clinical Research (2019, 2)
	Long-Term Follow-Up After Administration of Human Gene Therapy Products (Draft, 2020.1)	Guideline on quality, nonclinical and clinical aspects of medicinal products containing genetically modified cells (Draft, 2018.7)	Science Board: Reflection paper for genome-editing products (2020, 2)
Genome editing	No description on tools for genome editing	Products consisting of recombinant nucleic acid as an active ingredient and other components, and causing control, repair, replacement, insertion, or deletion of DNA sequences in humans	Modification of human genes of specific target DNA sequences and administration of genetically modified cells
Products including genome editing and raw materials	Gene therapy includes viral vectors and plasmids, as well as genome editing using mRNA	Vectors (including mRNA) coding for enzymes used for gene modification, enzyme proteins for gene modification, nucleic acid for genome editing, nucleic acid templates to be knocked in, and cells to be modified	Viral vectors, plasmids, mRNA, or proteins that modify the specific DNA sequences, nucleic acid, etc.

Off-target effects, on-target mutations due to DSB, chromosomal abnormalities, and immune reactions to genome-editing enzymes are pointed out by all three organizations as important issues related to safety. Currently there is no gold standard for identifying off-target modification. Orthogonal methods are used to identify potential off-target genome alteration and to quantify percentage of modification at a site. Not all genome modifications lead to deleterious biological consequences; however, assessment is limited due to lack of sensitive functional assays *in vitro* and *in vivo*. Unfortunately, animal models are of limited value for identifying and evaluating off-targets as the products are human genome specific. Importantly, since genome-editing therapy causes permanent genome changes, to evaluate potential tumorigenicity that may be caused by undesired genome changes or chromosomal abnormalities, long-term clinical follow-up should depend on the results of the preclinical characterization of the relevant genome-editing products.

Many new genome-editing technologies such as dead-Cas and based-Editing are still being developed. These new technologies also involve the risk of off-target effects, and studies on these effects and on undesired mutations are underway. Furthermore, a new approach to modifying the histone around target genes is being explored.^24^ It is not clear whether NRAs consider these technologies to be included in genome editing. It is clear that genome-editing technologies will continue to be developed one after another, and it is, therefore, difficult to determine what the focus of future genome editing might be. The characteristics and properties of genome editing must be revisited as necessary as the various technologies advance.

## Summary

This document summarizes recent discussions by experts in gene therapy and genome editing in Japan about the development of gene therapy products using genome-editing technology. We hope that these documents will help companies and researchers to develop new genome-editing-based gene therapy approaches, and assist reviewers in conducting regulatory reviews of genome-editing products. However, genome-editing technologies are advancing rapidly, and a great variety of technologies are emerging. This document should be revised as necessary to reflect technological advances.
